# Editorial: Control of cooperative drones and their applications

**DOI:** 10.3389/frobt.2022.1014510

**Published:** 2022-09-29

**Authors:** Rodrigo S. Jamisola

**Affiliations:** Department of Mechanical, Energy, and Industrial Engineering, Botswana International University of Science and Technology, Palapye, Botswana

**Keywords:** cooperative manipulation, unmanned aerial vehicles, cooperative control, systems communication, cooperative localization and mapping, cooperative navigation

**FIGURE 1:**
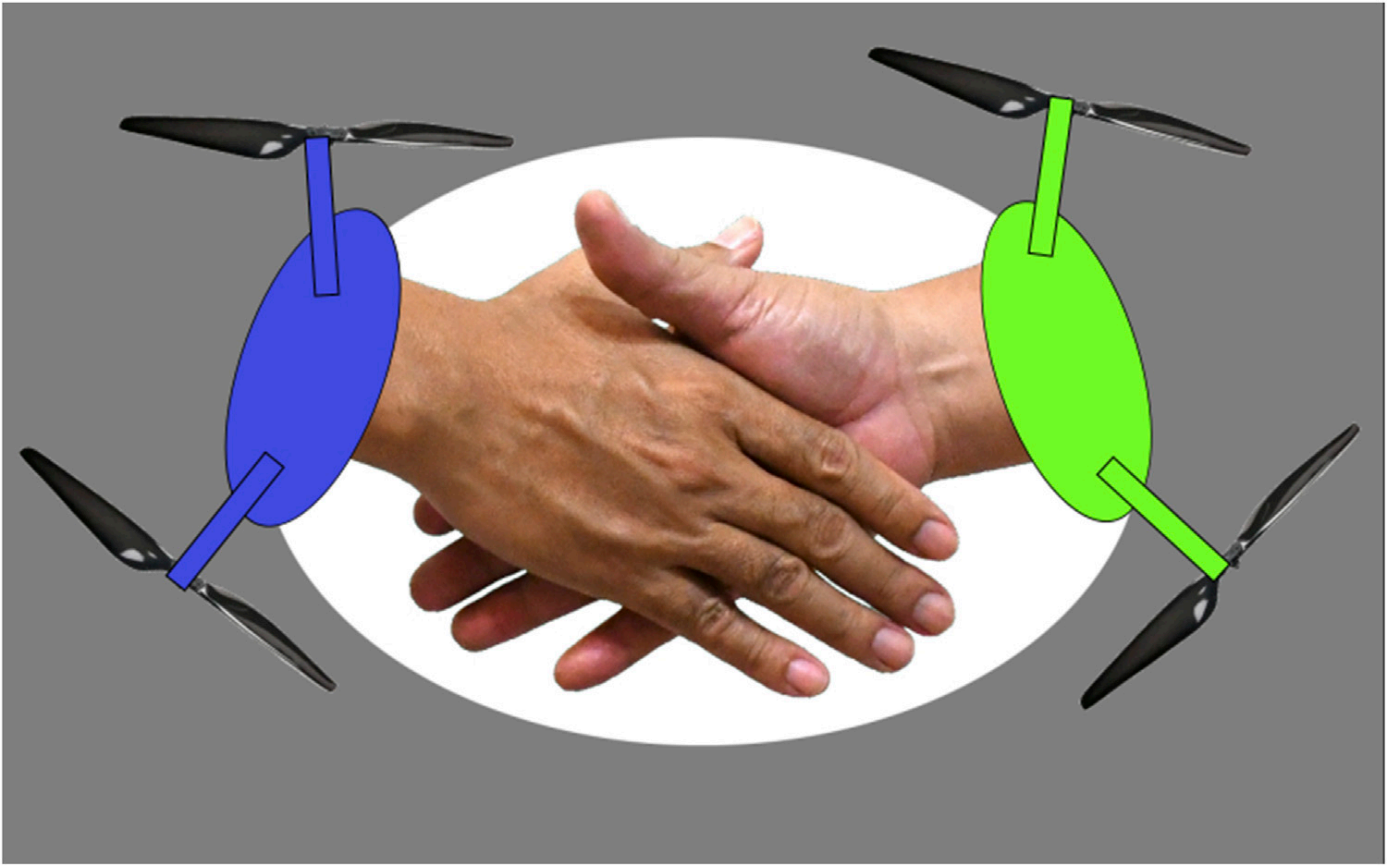
Drones cooperate to lighten the burden of the assigned task, just like when humans work together to achieve a common goal.

Cooperative drones alleviate the burden of a single drone to perform an assigned task, just like how humans cooperate and help each other towards a common goal. A depiction of drone cooperation is shown in Figure 1. They can afford faster execution, bigger coverage, larger payload, shared resources, and other increased capabilities not available in a single drone. However, this comes with a price in terms of control complexity, communication requirements, increased points of failure, cost of system components, and computational load. This Research Topic aims to present recent state-of-the-art solutions to pressing challenges faced by drones when cooperating to perform their common goal. These challenges can be critically understood when the solutions are focused on the common goal of cooperation which can be divided into three categories: shared load, shared task, and shared resource. The categories have overlaps but these classifications take on the highest priority goal of cooperation.

Shared load drone cooperation also includes cooperative manipulation, such that it encompasses drone cooperation that has direct contact with its environment. Most noteworthy are flying arms, which can be composed of dual-arms or more arms having “winged shoulders” capable of aerial dual-arm manipulation or several arms manipulation. This may revolutionize cooperative manipulation by the increased dimension of the workspace. In some cases, the mechanism attached to the drones may not be an arm, but just a gripper making the drone capable of grabbing objects. In this case, the task can be a simple cooperative pick-and-place or a cooperative perching of two or more drones to rest.

Shared task drone cooperation encompasses drone cooperation with no direct contact with the environment, but with a specific task to complete as a group. This includes increased area coverage in mapping, target tracking in large areas, target tracking with tracking size greater than individual drone coverage, multiple drone pesticide sprayers in agriculture, swarm drone light display, combating fire cooperatively, and many others. This type of drone cooperation is the most common and is expected to further increase significantly. In principle, the task capability of a single drone is simply multiplied such that the result is a collective task capability of the combined drones, but has no direct contact with the environment.

Lastly, shared resource drone cooperation puts more emphasis on the available resource of a single drone contributing to the combined available resource for all cooperating drones. This includes power sharing, energy harvesting, data integration, and communication relay. This kind of cooperation may be limited by the power requirement for the drones to keep afloat in the air but may be crucial in certain applications for a specific time.

There are four types of articles presented on this Research Topic: one article in Technology and Code, one Review article, and two Original Research articles. The first article develops a UAV software framework designed for application developers for multi-agent robotic systems. It characterizes an individual UAV and enables multi-agent communication and control systems, in a centralized and decentralized manner. It has three main features, namely, interoperability, distribution, and extensibility. It can run at different configurations, and multiple nodes, and can extend to different functions and other robotic platforms.

The Review article discusses state-of-the-art aerial robotic platforms with grasping and perching mechanisms for single aerial robots and multi-agent systems. It focuses on control and modeling for aerial manipulation mechanisms, particularly on the hardware and design innovations for aerial grasping and perching. One Original Research article investigates visual relative localization in a leader-follower formation with increased accuracy and minimized delay. Rather than relying on the autopilot data exchange, a visual scheme using passive markers is used. They are visually detected to determine the relative pose of the members and eliminate the need for RF communication.

Another Original Research article tackles multiple target tracking algorithms with targets moving in and out of field-of-view to improve the certainty of target estimates, including cooperating UAVs to increase coverage. The authors developed a method to incorporate road map information as well as a negative update to track multiple vehicles in an area larger than the drone’s sensing field of view in the presence of clutter and missed detections. Furthermore, it develops a novel method for maintaining target certainty by the tracker.

Overall, this edition of the Research Topic attempts to holistically provide the advancement of knowledge in cooperative drones through simulation platform software for multiple robots, a review on the hardware design for grasping in load sharing, a study on communication using vision for relative localization, and a study on tracking multiple targets in large areas.

Thus, the study areas presented here are critical components of drone cooperation to further advance its understanding. Other possible areas of further studies in drone cooperation are redundancy and fault tolerance; motion planning, localization and mapping; and endurance. The area of cooperative drones remains a vast rich field for exploration and discovery for researchers in this area of interest.

